# Ethyl-substitutive Thioflavin T as a highly-specific fluorescence probe for detecting G-quadruplex structure

**DOI:** 10.1038/s41598-018-20960-7

**Published:** 2018-02-08

**Authors:** Ai-jiao Guan, Xiu-Feng Zhang, Xin Sun, Qian Li, Jun-Feng Xiang, Li-Xia Wang, Ling Lan, Feng-Min Yang, Shu-Juan Xu, Xiao-Meng Guo, Ya-Lin Tang

**Affiliations:** 1 National Laboratory for Molecular Sciences, Center for Molecular Sciences, State Key Laboratory for Structural Chemistry of Unstable and Stable Species, CAS Research/Education Center for Excellence in Molecular Sciences, Institute of Chemistry Chinese Academy of Sciences, Beijing, 100190 P. R. China; 2 College of Chemistry Engineering, North China University of Science and Technology, Tangshan, 063009 P. R. China; 3 University of the Chinese Academy of Sciences, Beijing, 100049 P. R. China

## Abstract

G-quadruplex has attracted considerable attention due to their prevalent distribution in functional genomic regions and transcripts, which can importantly influence biological processes such as regulation of telomere maintenance, gene transcription and gene translation. Artificial receptor study has been developed for accurate identification of G-quadruplex from DNA species, since it is important for the G-quadruplex related basic research, clinical diagnosis, and therapy. Herein, fluorescent dye ThT-E, a derivative of the known fluorescence probe Thioflavin T (ThT), was designed and synthesized to effectively differentiate various G-quadruplex structures from other nucleic acid forms. Compared with methyl groups in ThT, three ethyl groups were introduced to ThT-E, which leads to strengthened affinity, selectivity and little inducing effect on the G-quadruplex formation. More importantly, ThT-E could be served as a visual tool to directly differentiate G-quadruplex solution even with naked eyes under illumination of ultraviolet light. Thus, this probe reported herein may hold great promise for high-throughput assay to screen G-quadruplex, which may widely apply to G-quadruplex-based potential diagnosis and therapy.

## Introduction

G-quadruplexes are non-canonical nucleic acids secondary structures which are consisted of guanine-rich nucleotide sequence via stacking of Hoogsteen hydrogen-bonded G-quartets in the presence of monovalent cations (usually K^+^ or Na^+^)^1,2^.Computational studies have revealed that G-quadruplex could exist simultaneously in human genome and transcriptome, such as chromosomal telomere ends, promoter, mitochondria genome and untranslated regions (UTRs) of mRNA^3–8^. Importantly, these structures are associated with a series of significant genome functions, including transcription, replication, recombination and maintenance of chromosome stability^9,10^. For instance, some G-quadruplex in promoter regions such as c-myc, c-kit, K-ras and VEGF are involved in regulating gene expression^11–13^. G-quadruplex in mRNA untranslated regions (UTRs) could regulate gene at the translation level^14–17^. Therefore, accurate recognition of G-quadruplex from other nucleic acid forms became pretty significant. So far, many approaches have been developed to identify G-quadruplex structure, such as fluorescence resonance energy transfer (FRET), circular dichroism (CD), nuclear magnetic resonance (NMR), X-ray crystallography^18–21^. Besides these techniques, recognizing G-quadruplex by fluorescent chemical probes may provide an efficient way for G-quadruplex screening and detecting. To date, many fluorescence “light-up” probes have been successfully designed and developed to recognize G-quadruplex^22–34^. These probes provide useful tools for *in vitro* and *in vivo* detecting G-quadruplex structures and regulating G-quadruplex related biological function^35–37^. Besides, fluorescent probes targeting G-quadruplex also used in the emerging G-quadruplex based label-free luminescence detection techniques^38–41^. However, in the reported G-quadruplex probes, most of them are water-insoluble and require complicated multi-step synthesis, which would limit their application in some case. Therefore, there is an urgent need for novel fluorescent probes targeting G-quadruplex structures.

**Figure 1:**
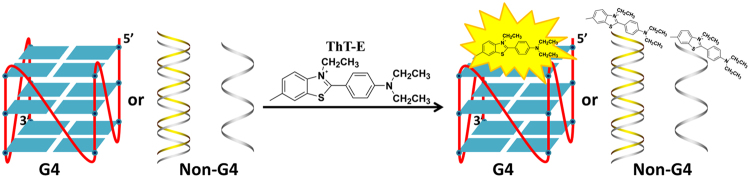
Schematic illustration for highly selective recognition of G-quadruplex based on the fluorescence light-up of ThT-E.

Thioflavin T (ThT), a commercially cationic benzothiazole dye, is served as a sensitive sensor for amyloid fibril and other tissue structures^42^. Recently, Mohanty *et al*. reported a detailed experimental and theoretical study of the interaction between ThT and human telomeric G-quadruplex, illustrating this probe could selectively recognize and stabilize G-quadruplex^43^. Furthermore, Mergny *et al*. expanded the studies to a series of oligonucleotides to further evidence that ThT could be used as a probe for efficient G-quadruplex sensing^44^. However, strong G-quadruplex-forming capacity induced by ThT can significantly change the topology of G-quadruplex, which may limit its application in dynamic detection of G-quadruplex structure formation. Consequently, it is highly desirable to design a new fluorescent probe that could recognize G-quadruplex with improved selectivity and little effect on the G-quadruplex formation. Recently, it has been witnessed that a larger side chain or a specific side chain of probes played important roles in improving the recognition of G-quadruplex through non-bond interactions, such as hydrogen bond, van der Waals force and others^45,46^. For example, the porphyrin derivatives with larger side chains have better selectivity towards G-quadruplex over duplex, which are more suitable for G-quadruplex sensing^47^. On the basis of the above information, we therefore conceived that a new ThT derivative with ethyl groups instead of methyl groups maybe better fit for G-quadruplex recognition. In this work, a new probe ThT-E was designed and synthesized via 1-step transformation starting from 2-(4-aminophenyl)-6-methylbenzothiazole (Fig. S1). Further studies indicated that the fluorescence of ThT-E enhance significantly when interacting with various G-quadruplex(Fig. 1), which enables ThT-E to be used as a highly-specific fluorescence sensor for G-quadruplex detection.

## Results and Discussion

### Design and Characterization of ThT-E

An attractive application of ThT is served as a sensor for G-quadruplex detection due to their selective fluorescence enhancement, which could be attributed to enforcing planarization and restricting rotation between benzothiazole and dimethylaminobenzene rings around the C-C bond^48^. Substituting methyl groups with ethyl of ThT may better for G-quadruplex recognition since larger side chains play important roles in improving the selectivity and affinity of ligand for G-quadruple^47^. To further evaluate the effect of ethyl groups on binding strength for G-quadruplexs, the G-quadruplex structure formed from oncogene c-myc promoter regions was selected to carry out molecular docking with ThT or ThT-E using G4LDB system^49^, respectively. The values of ∆G for ThT and ThT-E binding to the G-quadruplex structure were −27.57 kJ·mol^−1^ and −28.60 kJ·mol^−1^, respectively (Table S1). In comparison with ThT, the ThT-E showed higher binding ability to the parallel G-quadruplex structure. Obviously, these results demonstrated that ThT-E has a stronger binding strength than ThT for G-quadruplexs, implying that ThT-E may better differentiate G-quadruplexs from other nucleic acid forms. In this study, the water-soluble fluorogenic dye ThT-E was availably prepared via 1-step transformation starting from 2-(4-aminophenyl)-6-methylbenzothiazole and iodoethane with 61 percent productivity, indicating that the preparation is simple and inexpensive. The molar extinction coefficient of ThT-E is 49342 M^−1^cm^−1^, which was measured at λ = 418 nm in water (see Supplementary Fig. S2).

### ThT-E selectively targeted G-quadruplex by fluorescence light-up

**Figure 2:**
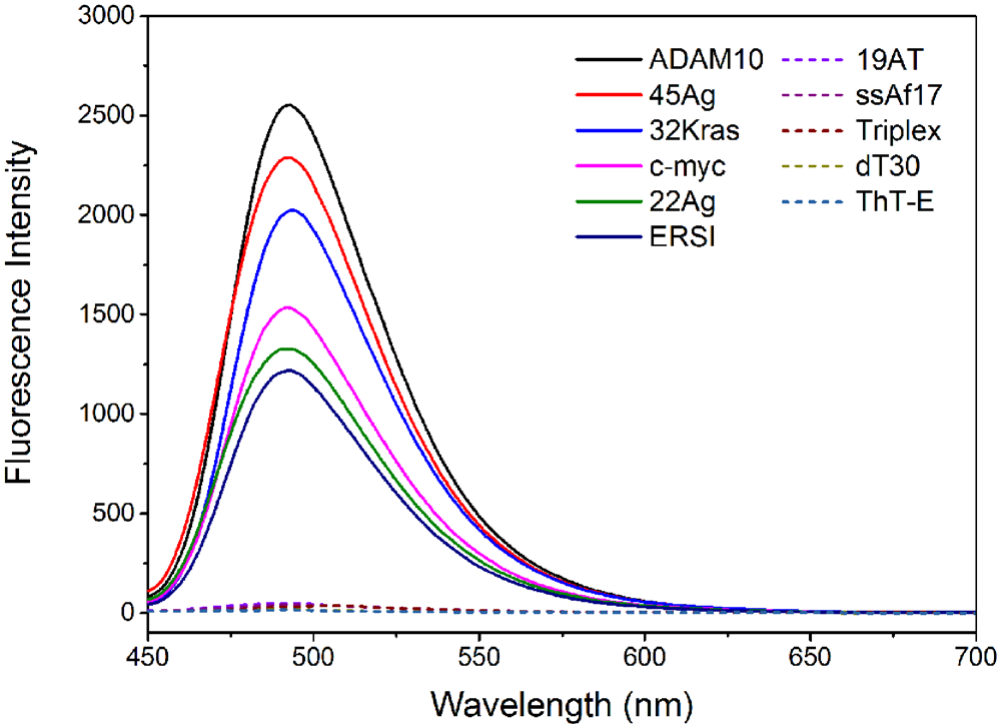
Fluorescence emission spectra of ThT-E (2 μM) with various oligonucleotides (2 μM) in a 20 mM Tris-HCl (40 mM K^+^, pH 7.4) solution.

To evaluate the feasibility of the ThT-E probe for G-quadruplex recognition, we compared the fluorescence intensity of ThT-E in the absence and presence of various DNA forms. As shown in Fig. 2, ThT-E alone in the Tris-HCl buffer exhibited negligible fluorescence emission. With the addition of G-quadruplexes, an emission peak at approximately λ = 492 nm appeared and remarkably enhanced. The F/F_0_ was determined to be 159-, 146-, 125-, 83-, 77- and 96-fold for ADAM10, 45Ag, 32Kras, 22Ag, ERSI and c-myc, respectively. In contrast, the fluorescence of ThT-E enhanced less than 3 fold in the presence of other DNA formations, such as duplex (19AT), single-strand (dT30, ssAf17) and triplex. Thus, ThT-E is an efficient candidate as a fluorophore to differentiate various G-quadruplex structures from other nucleic acid forms by fluorescence enhancement.

**Figure 3:**
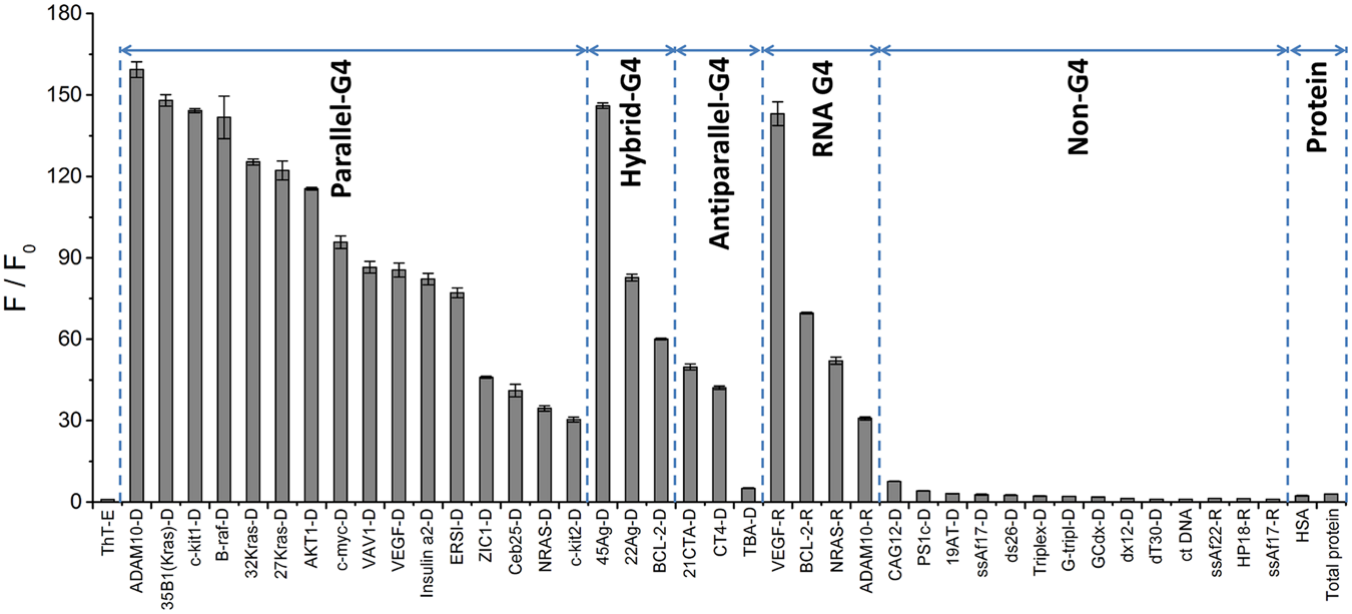
Diagrammatic bar representation of ThT-E (2 μM) fluorescence enhancement at 492 nm for a variety of oligonucleotide sequences (2 μM) or protein (HSA concentration is 2 μM, total protein concentration set as 20 mg/L) in a 20 mM Tris-HCl (40 mM K^+^, pH 7.4) solution. Error bars correspond to S.D.

To further certify the ability of selectivity recognition of ThT-E towards G-quadruplex, another 23 DNA sequences and 7 RNA sequences were introduced to interact with ThT-E. Moreover, the interactions between ThT-E and human serum albumin (HSA) or cell total protein (TP) were also inspected, respectively. As shown in Fig. 3, both DNA G-quadruplex and RNA G-quadruplex resulted in significant fluorescence enhancement of ThT-E. Interestingly, the B-raf bimolecular G-quadruplex induced remarkable fluorescence enhancement, indicating that ThT-E may has good recognition ability for high-order G-quadruplex. In contrast, much weaker fluorescence emission were observed when ThT-E interacting with TBA G-quadruplex and other nucleic acids forms. Importantly, ThT showed conspicuous fluorescence enhancement for some non-G-quadruplex structures, such as single-stranded (dT30), double-strands (PS1c) and triplex^44^. The fluorescence signals of ThT for these non-G-quadruplex structures even exceeded some G-quadruplex structures. This may lead to a false positive result when using the fluorescent probe ThT to recognize G-quadruplex structures. By contrast, the probe ThT-E does not emit significant fluorescence when interacting with the non-G-quadruplex structures. And it also emits strong fluorescence when interacting with G-quadruplex structures. As a result, the ThT-E could be used to accurately distinguish G-quadruplex and non-G-quadruplex structures. And ThT-E may not give such false positive results (dT30, PS1c and triplex) as the ThT does. Therefore, the fluorescent probe ThT-E shows much better ability for distinguish G-quadruplex and non-G-quadruplex structures than the ThT probe. On the other hand, HSA and TP only induced negligible fluorescence emission of ThT-E, implying the detection of G-quadruplex by ThT-E may not be affected by proteins. Owing to these results, we thus proposed that ThT-E could be served as a highly selective fluorescence “light-up” probe with promising recognition ability not merely for DNA G-quadruplex but for RNA G-quadruplex.

**Figure 4:**
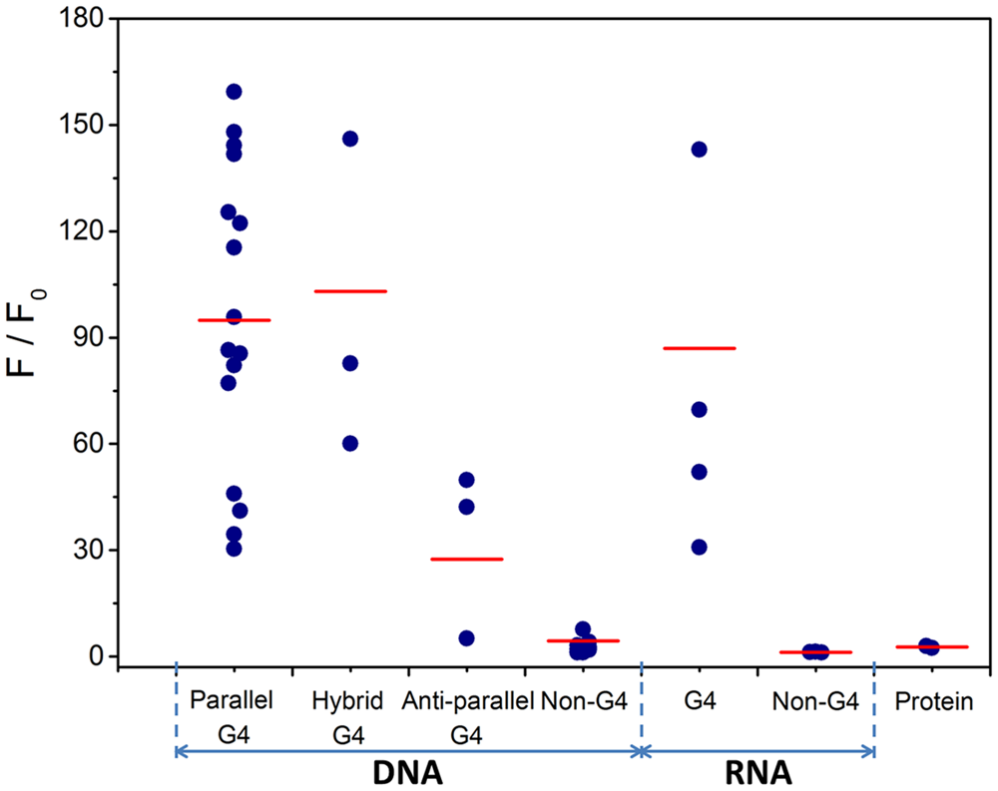
Scatter diagram of ThT-E (2 μM) fluorescence enhancement folds at 492 nm for a variety of oligonucleotide sequences (2 μM) or protein in a 20 mM Tris-HCl (40 mM K^+^, pH 7.4) solution.

Quadruplexes are structurally polymorphic and could be classified into parallel, antiparallel and hybrid types according to the strand polarities and location of the loops^19^. To further study the relationship between fluorescence behavior of ThT-E and G-quadruplex structure formation of these G-quadruplexes, we next carried out CD measurement (see Supplementary Fig. S3). As depicted in Fig. 4, the statistical data revealed that the fluorescence of ThT-E enhanced about 94.9-, 103-, 27.4- and 87-fold in the presence of parallel DNA G-quadruplex, hybrid DNA G-quadruplex, antiparallel DNA G-quadruplex and RNA G-quadruplex, respectively. Obviously, the enhancement of ThT-E fluorescence intensity for antiparallel G-quadruplexes is weaker than parallel or hybrid G-quadruplex structures, including 21CTA, TBA and CT4 sequences. Compared with ThT-E, there is no distinct rule in the ThT interacting with antiparallel G-quadruplex, such as ThT showed significant fluorescence enhancement for 21CTA G-quadruplex^44^. On the other hand, ThT-E fluorescence enhanced about 4.4-, 1.2- and 2.7 times in the presence of other nucleic acids forms and proteins. Moreover, competitive fluorescence titration assay was performed to further ascertain the selective recognition capacity of ThT-E for G-quadruplex. In this assay, ThT-E was titrated with the 22Ag in the presence of a large excess of duplex DNA (ct DNA, 50 μM bp). As shown in Fig. S4, the 22Ag induced similar fluorescence enhancement of ThT-E in the presence or absence of ct DNA, indicating that ThT-E holds great promise for G-quadruplex detection, even in a competitive biological environment.

### The fluorescent light-up effect can be detected even by naked eyes under ultraviolet light

**Figure 5:**
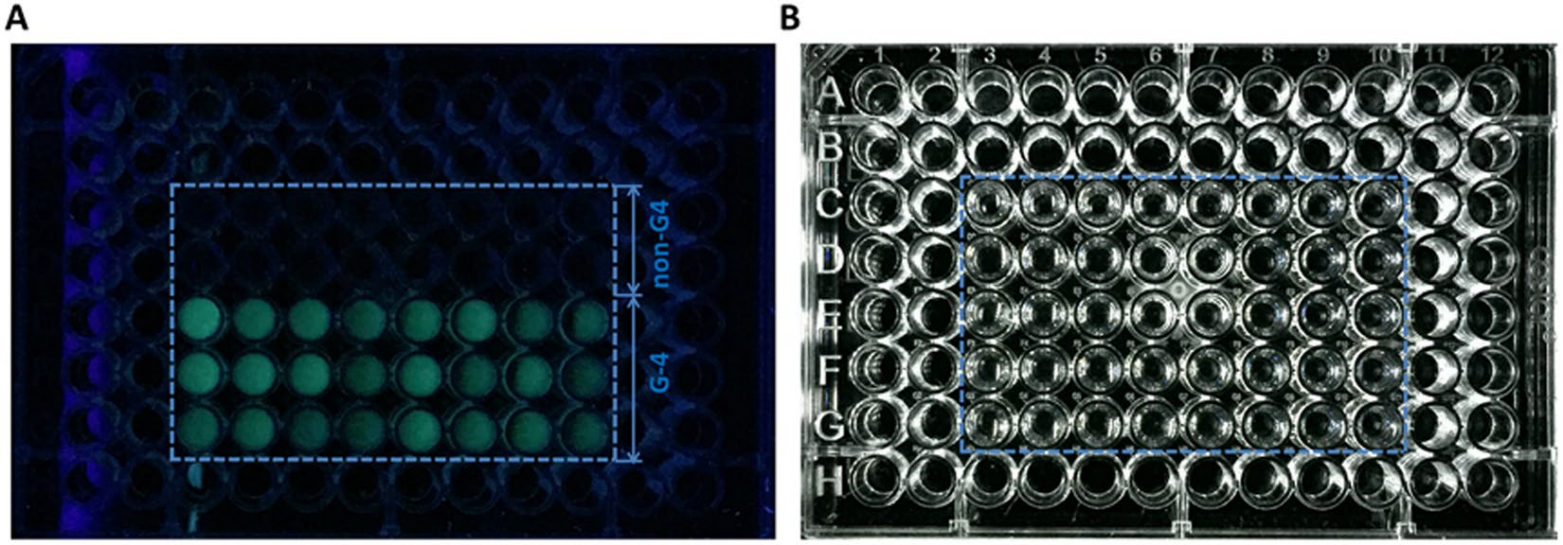
Photographs of 2 μM ThT-E (except for sample C4) with different oligonucleotides (2 μM) in20 mM Tris-HCl (40 mM KCl, pH 7.4) solution. (**A**) Ultraviolet light and (**B**) Visible light. C3 ~ C10: ThT-E, Tris-HCl buffer, CAG12-D, PS1c-D, 19AT-D, ssAf17-D, ds26-D, Triplex-D; D3 ~ D10: G-tripl-D, GCdx-D, dx12-D, dT30-D, ct DNA, ssAf22-R, HP18-R, ssAf17-R; E3 ~ E10: ADAM10-D, 35B1(Kras)-D, 32kras-D, 27Kras-D, AKT1-D, c-myc-D, VAV1-D, VEGF-D; F3 ~ F10: Insulin a2-D, ERSI-D, ZIC1-D, Ceb25-D, 45Ag-D, c-kit1-D, 22Ag-D, NRAS-D; G3 ~ G10: BCL-2Mid-D, c-kit2-D, 21CTA-D, CT4-D, BCL-2-R, VEGF-R, NRAS-R and ADAM10-R, respectively.

Based on the exact recognition of ThT-E to G-quadruplex under fluorescence spectra, we further explored the visual character of G-quadruplex in aqueous solution under ultraviolet light condition. As seen in Fig. 5, the difference in fluorescence resulting from the G-quadruplex and others can even be achieved with the naked eye under UV illumination. Almost all of the ThT-E solution in the presence of G-quadruplex exhibited an emission of greenish blue light with the aid of ultraviolet light (Fig. 5A). Meanwhile, the solution was transparent under other nucleic acid structures condition. On the other hand, there is no obvious distinction between G-quadruplex and non-G-quadruplex solutions under visible light, as shown in Fig. 5B. These results showed that the ThT-E could be an efficient fluorescent probe for high-throughput G-quadruplex detection and may be widely used in future G-quadruplex-based biomarker discovery.

### The ThT-E molecule can also selectively recognize G-quadruplex structures in the native polyacrylamide gel electrophoresis (PAGE) system

**Figure 6:**
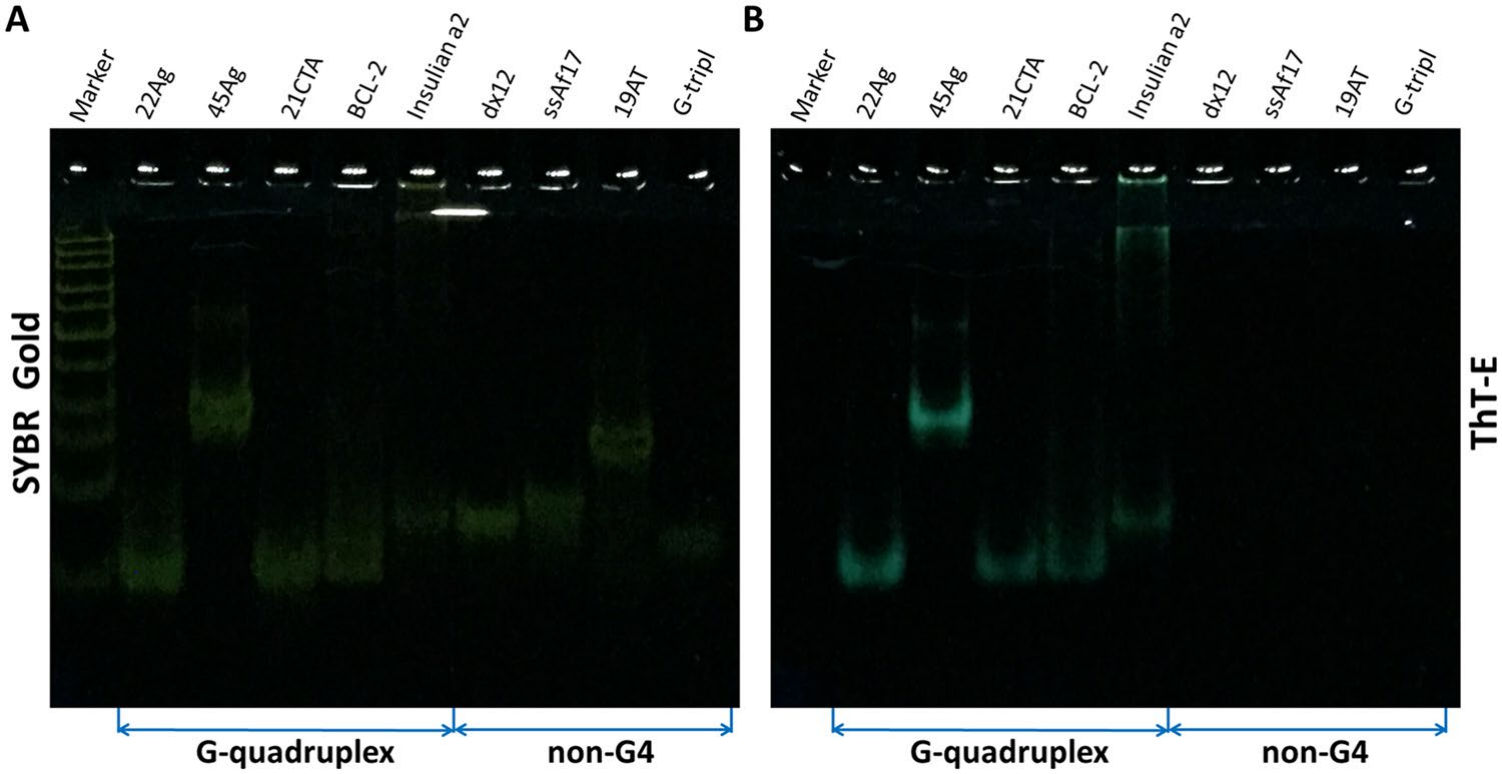
Selectivity assays on PAGE. DNA samples were prepared in a 20 mM Tris-HCl, pH 7.4, Loaded on a 5% stacking and 12% separating polyacrylamide gel. The gels were stained with (**A**) SYBR Gold, (**B**) 20 μM ThT-E, and visualized on a ZF-90 UV viewing cabinet.

The significant fluorescence enhancement of ThT-E when binding to G-quadruplex prompted us to investigate whether ThT-E could act as a G-quadruplex recognizer in electrophoresis gels. As shown in Fig. 6A, all bands could be detected after staining with SYBR Gold. In contrast, only the G-quadruplex 22Ag, 45Ag, 21CTA, BCL-2Mid and Insulin a2 could be detected after ThT-E staining (Fig. 6B). Compared to staining with SYBR Gold, the non-G-quadruplex dx12, ssAf17, 19AT and G-triplex did not show any band when staining with ThT-E. These results were in accord with the results from fluorescence and ultraviolet visualization experiments showing in Figs 3 and 5, implying ThT-E could also recognize G-quadruplex structures in the native gel system.

### UV-vis spectra showed that the presence of G-quadruplex could significantly induced red-shift of the ThT-E molecule

**Figure 7:**
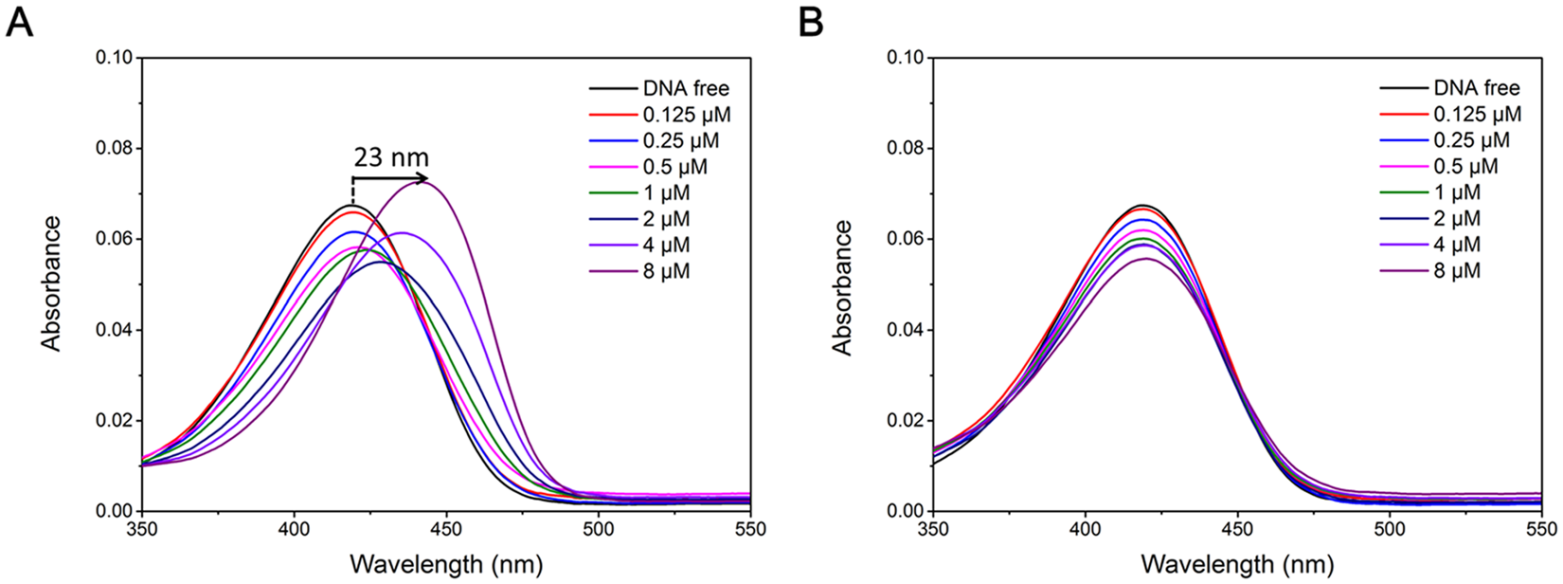
Absorbance spectra of ThT-E (2 μM) with DNA G-quadruplex sequence (**A**) c-myc (**B**) PS1c at eight concentrations (μM): (1) 0, (2) 0.125, (3) 0.25, (4) 0.5, (5) 1, (6) 2, (7) 4, (8) 8.

To get insight into the nature of the interactions between ThT-E and various nucleic acid sequences, absorption spectra was then applied to investigate the specific binding of ThT-E with G-quadruplex. Remarkably, ThT-E displayed distinguishing behaviors when interacting with different nucleic acid sequences. As shown in Fig. 7A, the absorption spectra showed an systematic red shift about 23 nm of the absorption band, accompanied a strong hypochromicity and hyperchromicity with gradual addition of the G-quadruplex DNA from 0 to 4 equivalent. In addition, as shown in Fig. 7B, the non-G-quadruplex (with PS1c duplex as an example) only showed moderate red shift and hypochromic effect, implying the weaker interaction between non-G-quadruplex and ThT-E. These distinctions of absorbance spectra between G-quadruplex and non-G-quadruplex may attribute to the different interaction modes between ThT-E and DNA. It has been reported that the interaction of organic dyes with ds-DNA or single strand DNA mainly through intercalative, electrostatic or groove binding modes^50,51^. However, the electron-rich aromatic core of ThT-E can interact with G-quartet through π-π stacking mode at the end of G-quadruplex. Simultaneously, the side arm ethyl groups may also interact with loops or bind to the grooves of the G-quadruplex.

### The ThT-E molecule may also detect non-canonical G-quadruplex structures by fluorescence light-up effects

**Figure 8:**
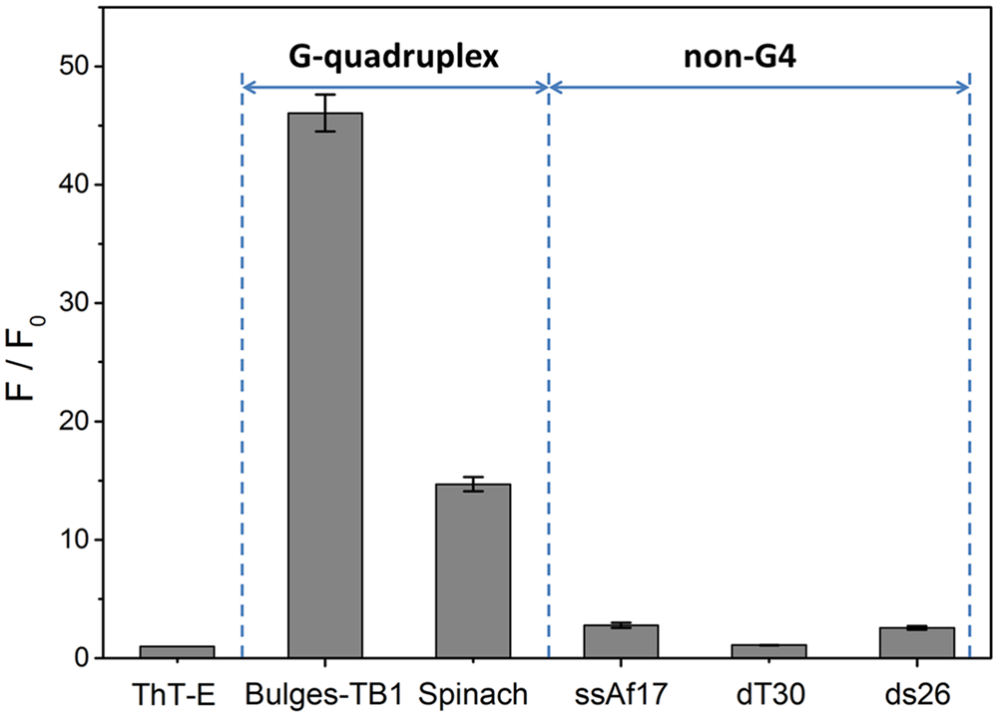
Dependence of ThT-E (2 μM) fluorescence intensity on non-canonical G-quadruplex and non-G-quadruplex sequences in a 20 mM Tris-HCl (40 mM K^+^, pH 7.4) solution.

The criteria for potential G-quadruplex sequence was restricted to: G_3-5_N_L1_G_3-5_N_L2_G_3-5_N_L3_G_3-5_^52^. However, some sequences defying this standard notion could still form stable G-quadruplex structure, such as Bulges-TB1 and Spinach^53,54^. In addition, there are fewer reports about G-quadruplex probe to availably detect these non-canonical G-quadruplex structures. To explore the suitability of ThT-E for these G-quadruplexes, the non-canonical G-quadruplex Bulges-TB1 and Spinach were introduced to evaluate the recognition ability of ThT-E. As shown in Fig. 8, the fluorescence intensity of ThT-E increased about 46.05- and 14.71-fold in the presence of Bulges-TB1 and Spinach, respectively. On the contrary, single-strand DNA (ssAf17 and dT30), double-strand (ds26) only induced 2.77-, 1.11- and 2.56-fold, respectively. Obviously, the fluorescence induced by non-canonical G-quadruplex is much higher than non-G-quadruplex structures. From these results, we infer that ThT-E may be suitable for the extensive detection of non-canonical G-quadruplex structures.

### The ThT-E probe showed higher binding affinity with parallel G-quadruplex but similar binding affinity with hybrid G-quadruplex structure compared to ThT

**Figure 9:**
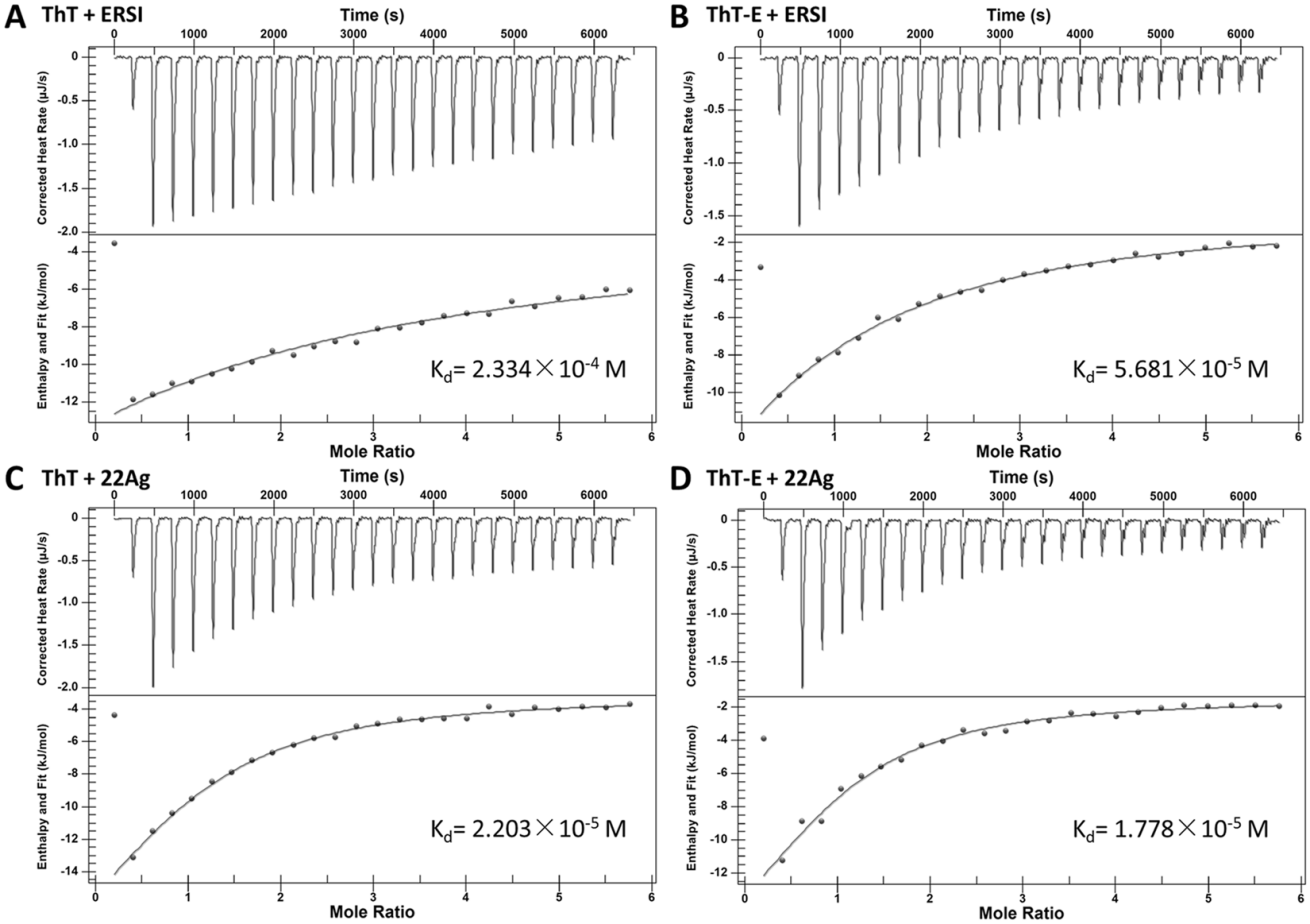
Representative results of G-quadruplexes with ThT (**A**,**C**) or ThT-E (**B**,**D**). Calorimetric analysis of the interaction of the G-quadruplexs (22Ag and ERSI) with the ThT or ThT-E.

In order to design a fluorescent probe targeting G-quadruplex with higher selectivity and affinity, we rationally designed and synthesized the fluorescent probe ThT-E. The isothermal titration calorimetry (ITC) was utilized to investigate the binding affinity between G-quadruplexs and ThT or ThT-E. As to the ThT-E molecule, a 1:1 binding model was performed, which has been confirmed by a Job’s plot analysis in this study (see Supplementary Fig. S5). As shown in Fig. 9, the dissociation constants of ThT and ThT-E for parallel-G-quadruplex ERSI are 2.334 × 10^−4^ M and 5.681 × 10^−5^ M, respectively. Meanwhile, ThT and ThT-E showing a dissociation constant for parallel-G-quadruplex VAV1 are 3.344 × 10^−4^ M and 7.473 × 10^−5^ M, respectively (Fig. S6). Based on the above information, we therefore concluded that the ThT-E probe may be more favorable to bind to parallel G-quadruplex than ThT. On the other hand, ThT and ThT-E exhibited similar binding affinity for hybrid- G-quadruplex 22Ag with dissociation constants of 2.203 × 10^−5^ M and 1.778 × 10^−5^ M, respectively. The above results were highly in agreement with the results from molecular docking data and significant enhancement of fluorescence upon the ThT-E interacting with parallel and hybrid G-quadruplexes. In previous study, it has been confirmed that ThT could selectively target G-quadruplex with hybrid structures through its fluorescence light-up^48^. As a result, we infer that the ThT-E may be more favorable to bind to parallel G-quadruplex than ThT and may be used as a better fluorescent probe for G-quadruplex sensing.

### The ThT-E molecule showed much less ability to induce the putative G-quadruplex sequences to form G-quadruplex structures than ThT in the absent of monovalent cations

**Figure 10:**
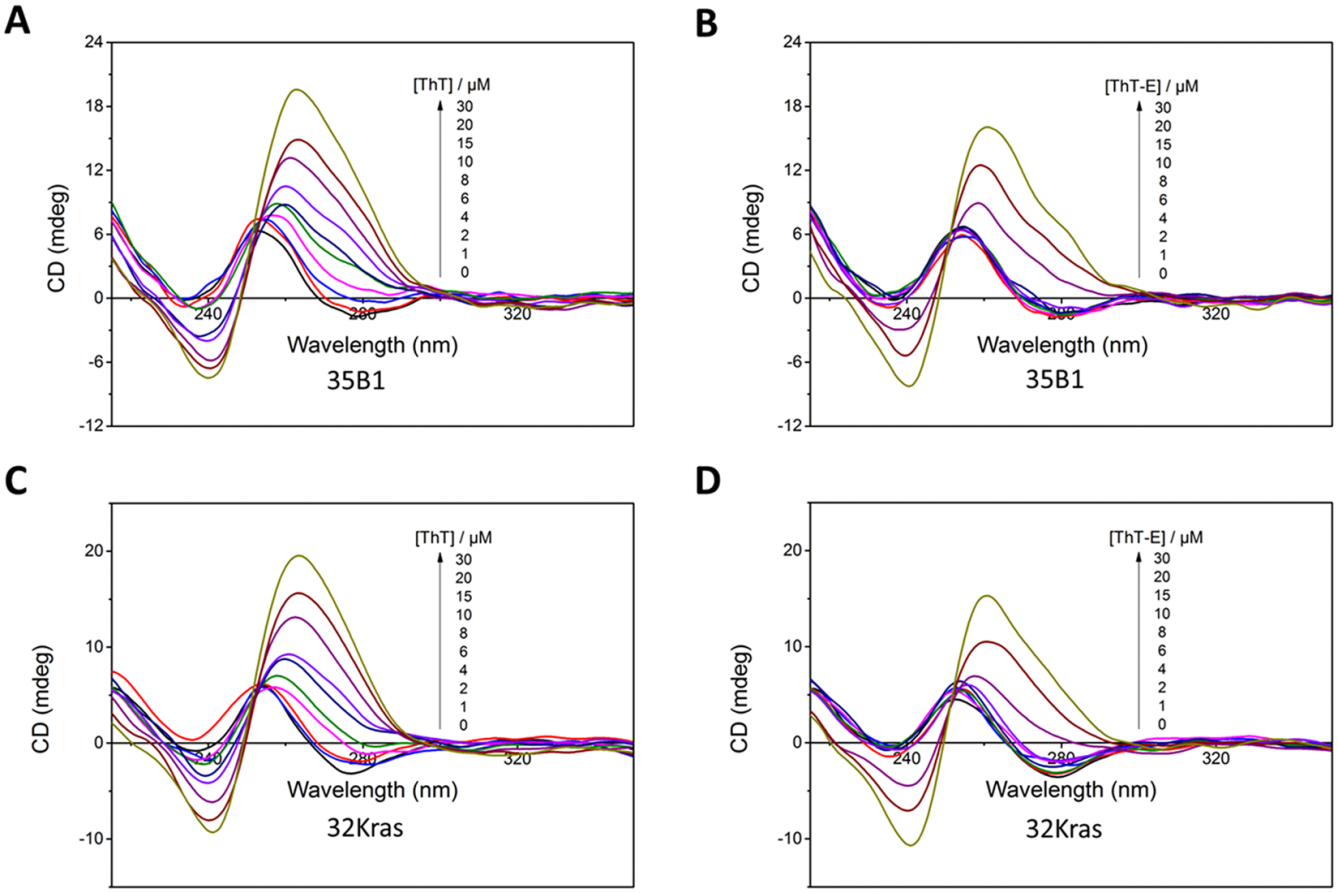
Circular dichroism spectra of 35B1 (**A**,**B**) and 32Kras (**C**,**D**) DNA sequences (2 μM) at 20 mM Tris-HCl (pH 7.4) solution with ThT (**A**,**C**) or ThT-E (**B**,**D**) of concentrations (μM): 0, 1, 2, 4, 6, 8, 10, 15, 20, 30.

ThT as an efficiently selective fluorescence probe for G-quadruplex versus other DNA structure holds great promise for potential diagnostic applications. However, the ThT molecule could significantly induce the putative G-quadruplex sequences to form G-quadruplex structure even in the absence of monovalent cations. That means the ThT molecule itself may affect the sequence’s structure and may limit its application in dynamic detection of G-quadruplex structure formation. In order to evaluate the probe’s effect on formation of G-quadruplex, ThT and ThT-E were introduced to interact with two putative G-quadruplex sequences (35B1 and 32Kras) in the absence of metal ions, respectively. As shown in Fig. 10, both 35B1 and 32Kras displayed a positive band at 255 nm in 20 mM Tris-HCl (pH 7.4) buffer solution, corresponding to the CD feature of unfolded structure^43^. With increasing the concentration of ThT from 0 to 30 μM, the CD spectra gradually changed to a negative band at 240 nm and a positive band at 265 nm, corresponding to the CD features of parallel G-quadruplex. Again, ThT had been confirmed that it could induce the putative G-quadruplex sequences to form G-quadruplex structures in the absent of salt. However, there was no significant change of CD bands when the concentration of ThT-E is less than 10 μM, implying a weaker G-quadruplex-inducing ability of ThT-E probe. That is, ThT-E has no effect on the structure of G-quadruplex when the concentration of ThT-E is lower than 10 μM. Furthermore, UV illumination measurement was then applied to study the inducing ability of ThT or ThT-E for potential G-quadruplex formation sequences in the absence of monovalent cations. As seen in Fig. S7, the solutions of 32Kras and 35B1 exhibited transparent in the presence of ThT-E, confirming that the oligonucleotides still maintained its unfolding state. That is, ThT-E has a weaker ability to induce the structure’s change of nucleic acids. In contrast, both 32Kras and 35B1 solutions exhibited greenish blue in the presence of ThT condition under UV illumination, which may attribute to the transition of DNA formation from random unfolded state to G-quadruplex structure. Based on above results, we therefore proposed that ThT-E has much less structure’s effect of putative G-quadruplex sequences than ThT. Therefore, the ThT-E probe is more suitable for dynamically detecting the G-quadruplex’s folding states. Overall, these results would facilitate to understand the behavior of ThT-based probe interacting with G-quadruplexes and provide a good structural thought and basis to design or develop G-quadruplex probes and drugs.

### ^1^H-NMR results showed that the ThT-E molecule most probably binding to the G-quadruplex at the 5′-end G-tetrad

**Figure 11:**
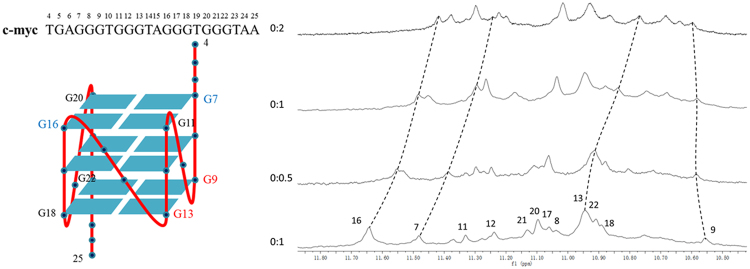
The schematic illustration of the c-myc G-quadruplex (left). And the imino proton regions of the ^1^H NMR titration spectra (10.5–11.8) of c-myc in pH = 7.4 40 mM K^+^ PBS solution with varying the [ThT-E]: [c-myc G-quadruplex] ratio (right).

To gain further insights into the interaction of G-quadruplex with ThT-E, 1D ^1^H NMR titration experiment were performed with various concentrations of c-myc. As shown in Fig. 11, there are 12 well-resolved imino proton peaks around 10.5–11.8 ppm, which comes from the tetrad-guanines of three G-tetrads of c-myc. All assignments of the imino proton peaks have been done according to Yang’s work^55^. With addition of ThT-E, these peaks showed significant changes in the shape and position, indicating the interaction occurred between ThT-E and G-tetrads. As shown in Fig. 11, the guanines G7, G11, G16 and G20 constitute the 5′-end G-tetrad of c-myc G-quadruplex, and G9, G13, G18, G22 make up the G-tetrad which locates in the 3′-end of G-quadruplex. Further analysis revealed that the change of chemical shift of G7 and G16 (locate in the 5′-end G-tetrad) are more prominent than G9 and G13 (locate in the 3′-end G-tetrad) (Table S2), implying the ThT-E may stack to the 5′-end G-tetrad of the G-quadruplex.

## Conclusions

To conclude, we designed and synthesized a new fluorescent dye ThT-E via 1-step reaction starting from 2-(4-aminophenyl)-6-methylbenzothiazole. The ThT-E was particularly attractive for distinguishing G-quadruplex (DNA and RNA, canonical and non-canonical) from other nucleic acid forms with high selectivity and sensitivity. The G-quadruplex solution could also be visualized even by naked eyes under ultraviolet. Compared with ThT, ThT-E showed higher binding affinity, selectivity for G-quadruplex and little effect on the structure of G-quadruplex forming sequences, which is more suitable for G-quadruplex detection. Overall, the synthesis of ThT-E and assay in this study may be the first step for the G-quadruplex based bio-analysis, further studies for broadening the application of this probe are now underway.

## Materials and Methods

### Molecular mechanism screening

Molecular docking was carried out using G4LDB system (http://www.g4ldb.org). The “compute” function was chosen to predict the binding strength between G-quadruplexs and ThT or ThT-E. The molecular docking and binding evaluation of four intra-G-quadruplexs with ThT or ThT-E were performed automatically after designing the ligand and submitting to G4LDB server.

### Oligonucleotides and proteins

Oligonucleotide sequences were listed in Table 1. The DNA sequences were purchased from Zixi Bio Tech Co., Ltd (Beijing, China) and RNA sequences were synthesized by Ribo Bio Co., Ltd (Guangzhou, Guangdong, China). Human serum albumin (HSA) and calf thymus DNA (ct-DNA) were purchased from sigma and used without further purification. Total protein (TP) was extracted from HL60 cell by protein extraction kit (BestBio. Co. Ltd. Shanghai, China) and then quantified via BCA protein assay kit (BestBio. Co. Ltd. Shanghai, China). Oligonucleotides and proteins were dissolved directly in 20 mM Tris-HCl buffer (40 mM KCl, pH = 7.4). Before testing, all oligonucleotides were heated to 90 °C for 5 min following with slowly cooling to room temperature. Ultrapure water was prepared with deionized water purified by Milli-Q Gradient ultrapure water system (Millipore) and used throughout all experiments.

**Table 1:** Oligonucleotides used in this work.

Name	Type/origin	Sequence (from 5′ to 3′)	Ref
22Ag-D	G4-HumanDNA telomere	AG_3_TTAG_3_TTAG_3_TTAG_3_	^43^
45Ag-D	G4-Human DNA telomere	G_3_TTAG_3_TTAG_3_TTAG_3_TTAG_3_TTAG_3_TTAG_3_TTAG_3_	
21CTA-D	G4-Human telomere variant	G_3_CTAG_3_CTAG_3_CTAG_3_	^56^
c-myc-D	G4-Promoter	TGAG_3_TG_3_TAG_3_TG_3_TAA	^57^
c-kit1-D	G4-Promoter	G_3_AG_3_CGCTG_3_AGGAG_3_	^58^
B-raf-D	G4-Promoter	G_3_CG_4_AG_5_AAG_3_A	^59^
c-kit2-D	G4-Promoter	G_3_CG_3_CGCGAG_3_AGG_3_	
TBA-D	G4-Aptamer	G_2_TTG_2_TGTG_2_TTG_2_	
BCL-2Mid-D	G4-Promoter	G_3_CGCG_3_AG_2_AATTG_3_CG_3_	^60^
Insulin a2-D	G4-Promoter	ACAG_4_TGTG_4_ACAG_4_TGTG_4_	^61^
AKT1-D	G4-Promoter	G_3_CG_3_CGGCTCCG_3_CGCG_3_	
VEGF-D	G4-Promoter	G_3_AG_3_TTG_4_TG_3_	^62^
VAV1-D	G4-Promoter	G_3_CAG_3_AG_3_AACTG_3_	
35B1(Kras)-D	G4-Promoter	AG_3_CGGTGTG_3_AAGAG_3_AAGAG_5_AG_2_CAG	
32 Kras-D	G4-Promoter	AG_3_CGGTGTG_3_AAGAG_3_AAGAG_5_AGG	^63^
27 Kras-D	G4-Promoter	G_3_CG_2_TGTG_3_AAGAG_3_AAGAG_4_	
Ceb25-D	G4-Minisatellites	AG_3_TG_3_TGTAAGTGTG_3_TG_3_T	
CT4-D	G4/Mixed quartets	G_3_CT_4_G_3_C	
ZIC1-D	G4–5′-UTR	G_3_TG_8_CG_5_AG_2_CCG_4_	
NRAS-D	G4–5′-UTR	G_3_AG_4_CG_3_TCTG_3_	
ADAM10-D	G4–5′-UTR	G_5_ACG_3_TAG_4_CG_3_AG_2_TAG_4_	^64^
ERSI-D	G4–5′-UTR	G_3_TAG_4_CAAAG_4_CTG_4_	
ssAf17-D	Single strand	CTGAGTTGTATATATTCG	
NRAS-R	G4–5′-UTR	G_3_AG_4_CG_3_UCUG_3_	^65^
Bulges-TB1	Non-canonical G4	TTGTG_2_TG_3_TG_3_TG_3_T	^53^
Spinach	Non-canonical G4	GCAGCCG_2_CTTGTTGAGTAGAGTGTGAGCT	^54^
		CCGTAACTG_2_TCGCGTC	
VEGF-R	G4-Promoter	G_2_AG_2_AG_5_AG_2_AG_2_A	^66^
NRAS-R	G4–5′-UTR	G_3_AG_4_CG_3_UCUG_3_	^67^
ADAM10-R	G4–5′-UTR	G_5_ACG_3_UAG_4_CG_3_AG_2_UAG_4_	^64^
BCL-2-R	G4–5′-UTR	AG_5_CCGUG_4_UG_3_AGCUG_4_	^68^
CAG12-D	Trinucleotide	(CAG)_12_	^69^
ds26-D	Duplex	CAATCG_2_ATCGAATTCGATCCGATTG	
G-tripl-D	Single strand	GGTTGGTGTGG	
		(a) TTTTTTTTTTTTTTTTTTTT	
Triplex-D	Triplex	(b) AAAAAAAAAAAAAAAAAAAA	
		(c) TTTTTTTTTTTTTTTTTTTT	
dT30-D	Single strand	T_30_	
GCdx-D	Stem-loop	GCGCGCGCT_4_GCGCGCGC	
PS1c-D	Parallel-duplex	(a) TTTTTTTTTTATTAAAATTTATAA	
		(b) AAAAAAAAAATAATTTTAAATATT	
19AT-D	Duplex	(a) ACGTCGATTATAGACGAGC	
		(b) GCTCGTCTATAATCGACGT	
dx12-D	Duplex	(a) GCGTGAGTTCGG	
		(b) CCGAACTCACGC	
ssAf22-R	Single strand	UGAGCUUAAUUGUAUAUAUUCG	
HP18-R	Hairpin	CAGUACAGAUCUGUACUG	^70^
ss-Af17-R	Single strand	CUGAGUUGUAUAUAUUCG	

### Synthesis of the ThT-E

To a sealed tube that contained a stir bar were added 2-(4-aminophenyl)-6-methylbenzothiazole (1.2 g, 5 mM), iodoethane (2.34 g, 15 mM) and potassium carbonate (1.38 g, 10 mM). The resulting mixture was stirred and heated at 140 °C for 14 h. Upon completion of the reaction, the mixture was cooled to room temperature and concentrated with the aid of a rotary evaporator. And the residue was purified by column chromatography on silica gel (the percent of methanol in dichloromethane from 2% to 20%). The pure product was isolated as a yellow solid (1.38 g, 61 percent). 2-(4-Aminophenyl)-6-methylbenzothiazole and iodoethane were obtained from J&K Company, Ltd. Dichloromethane and methanol were obtained from Beijing Chemical Plant (Beijing, China). 1H NMR a(400 MHz, CD_3_OD): 7.98–7.96 (d, 1H), 7.93 (s, 1H), 7.69–7.6 (d, 2H), 7.63–7.61 (d, 1H), 6.92–6.90 (d, 2H), 4.72–4.66 (q, 4H), 3.51–3.46 (q, 4H), 2.50 (s, 3H), 1.65 (t, 3H), 1.17 (t, 6H). 13CNMR (500 MHz, CD3OD): δ 174.95, 153.83, 141.64, 140.57, 133.41, 132.38, 130.42, 124.49, 117.40, 113.38, 111.90, 47.21, 45.93, 21.53, 14.85, 12.80; ESI-MS (m/z): [M]^+^ calcd. for C_20_H_25_N_2_S^+^, 325.17; found, 325.2. Analysis (calcd., found for C_20_H_25_N_2_S^+^): C (53.10, 52.26), H (5.57, 5.57), N (6.19, 5.91).

### Fluorescence spectroscopy

Fluorescence spectra were recorded with a Hitachi F-4600 spectrophotometer in a 1-cm path-length quartz cell with excitation of 420 nm and emission wavelength of 492 nm at room temperature. Both excitation and emission slits were 5 nm and voltage was 700 V and the scan speed was set as 1200 nm/min.

### UV-vis measurements

The absorption spectra were performed on an Agilent 8453 UV-vis spectrophotometer with a 10 mm light path cuvette at room temperature. The absorption titration experiments were carried out by increasing the concentration of oligonucleotide sequences from 0.125 to 8 μM, and ThT-E concentration was fixed at 2 μM.

### Circular dichroism spectroscopy

CD spectra were carried out on a Jasco-815 spectrometer equipped with a 10-mm path-length quartz cuvette at 298 K in the wavelength range of 200–500 nm. The scan speed was set as 500 nm/min and the response time was 0.5 ns. Each spectrum was the average of three scans.

### Visual observation

Photographs of ThT-E in the presence of series oligonucleotide sequences were captured in ZF-90 UV viewing cabinet with ultraviolet light or visible light. The concentrations both oligonucleotide and ThT-E of solution were fixed at 2 μM.

### Polyacrylamide gel electrophoresis

Gel electrophoresis was performed with a 5% stacking and 12% separating polyacrylamide gel. 2.5 μL various oligonucleotides (30 μM for ThT-E, 5 μM for SYBR Gold) were run in 2 × TBE buffer (0.18 M Tris-boric acid and 4 mM EDTA). Electrophoresis was run at 65 V for 90 min at room temperature. After electrophoresis, the gels were post-stained with a solution of 1 × SYBR Gold or 20 μM ThT-E in aqueous solution for 10 min, and then briefly washed in water before visualization on a ZF-90 UV viewing cabinet. The polyacrylamide gels were photographed by a digital camera under the aid of ultraviolet light and visible lights.

### Isothermal titration calorimetry

Calorimetric measurements were carried out using Nano ITC 2G Isothermal Titration Calorimete. The DNA, ThT and ThT-E stock solution were prepared by dissolving the with 20 mM Tris-HCl buffer (40 mM K^+^, pH = 7.4). In each experiment, volumes of 10 μL of a 500 μM ThT (ThT-E) solution were injected in a 25 μM DNA solution, with a spacing of 250 s between each injection.

### NMR experiments

The oligonucleotide was dissolved in phosphate buffer (40 mM K^+^, 90% H_2_O/10% deuteroxide). The ThT-E was firstly dissolved in water as a 5 mM stock solution, and then the measured samples were obtained by adding ThT-E stock solution into c-myc solution. The final concentration of c-myc was 1 mM. All NMR spectra were carried out on a Bruker Avance 600 spectrometer equipped with a 5 mm BBI probe at 25 °C.

## Electronic supplementary material

Supporting Information
