# Supplementary material for: Ethyl-substitutive Thioflavin T as a highly-specific fluorescence probe for detecting G-quadruplex structure
Source: Sci Rep. 2018 Feb 8;8:2666. doi: 10.1038/s41598-018-20960-7 (PMC5805748; doi:10.1038/s41598-018-20960-7)
Supplement: Supplementary file 1 — Supporting Information [file 41598_2018_20960_MOESM1_ESM.pdf]

Supplementary Information

Ethyl-substitutive Thioflavin T as a highly-specific fluorescence probe for detecting G-quadruplex structure

Ai-jiao Guan[1], Xiu-Feng Zhang[2], Xin Sun[2], Qian Li[1,3,*], Jun-Feng Xiang[1,3], Li-Xia Wang[1,3,*], Ling Lan[2,3], Feng-Min Yang[1,3], Shu-Juan Xu[1,3], Xiao-Meng Guo[1,3], Ya-Lin Tang[1,3,*]

1 National Laboratory for Molecular Sciences, Center for Molecular Sciences, State Key Laboratory for Structural Chemistry of Unstable and Stable Species, CAS Research/Education Center for Excellence in Molecular Sciences, Institute of Chemistry Chinese Academy of Sciences, Beijing, 100190, P. R. China.

2 College of Chemistry Engineering, North China University of Science and Technology, Tangshan, 063009, P. R. China.

3 University of the Chinese Academy of Sciences, Beijing, 100049, P. R. China.

§ These authors contributed equally to this work.

* To whom correspondence should be addressed: tangyl@iccas.ac.cn, qianlee@iccas.ac.cn, wlx8825@iccas.ac.cn;Tel: + 86 10 62522090; Fax: + 86 10 62522090.

Contents

**Part 1**	**G4LDB docking**
**Part 2**	**Synthesis of ThT-E**
**Part 3**	**Molar extinction coefficient of ThT-E**
**Part 4**	**Circular dichroism of DNA G-quadruplexes**
**Part 5**	**Spectrofluorimetric titration of the 22Ag**
**Part 6**	**Job's plot analysis**
**Part 7**	**Determination of dissociation constants by ITC**
**Part 8**	**UV illumination measurement**
**Part 9**	**The chemical shift's changes of the peaks of imino protons**
**Part 10**	**Identification of ThT-E**

Part 1 G4LDB docking

Table S1. G4LDB docking results for ThT and ThT-E

22-b	2L7V	d(TGAGGGTGGGTAGGGTGGGTAA)
Probe	ThT	ThT-E
Docking results	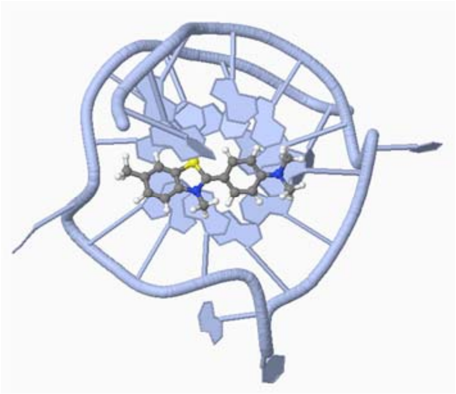	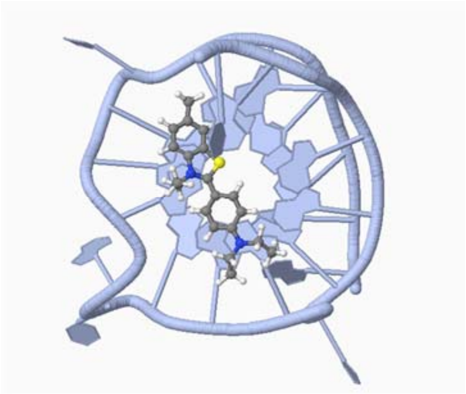
ΔG	-27.57 kJ·mol-1	-28.60 kJ·mol-1

Part 2 Synthesis of ThT-E

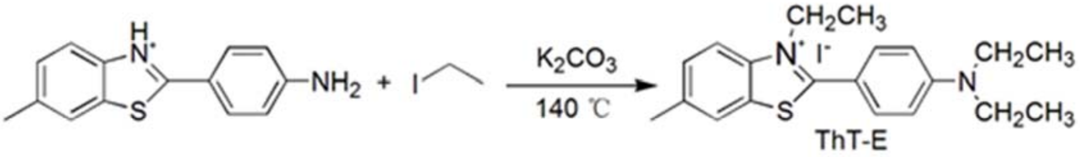

Figure S1. Synthesis of ThT-E.

Part 3 Molar extinction coefficient of ThT-E

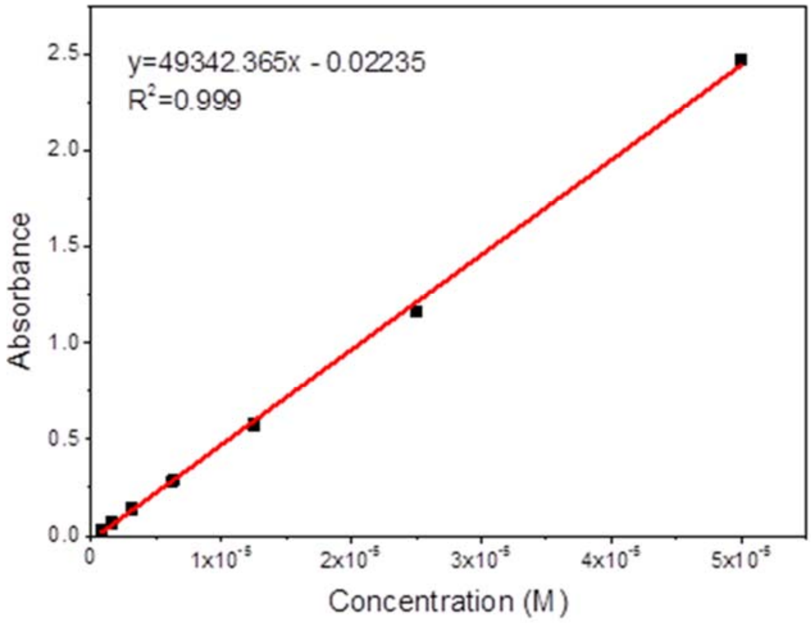

Figure S2. Dependence absorbance of ThT-E solution at 418 nm in seven concentrations (μM): (1) 0.78125 (2) 1.5625, (3) 3.125, (4) 6.25, (5) 12.5, (6) 25, (7) 50.

Part 4 Circular dichroism of DNA G-quadruplexes

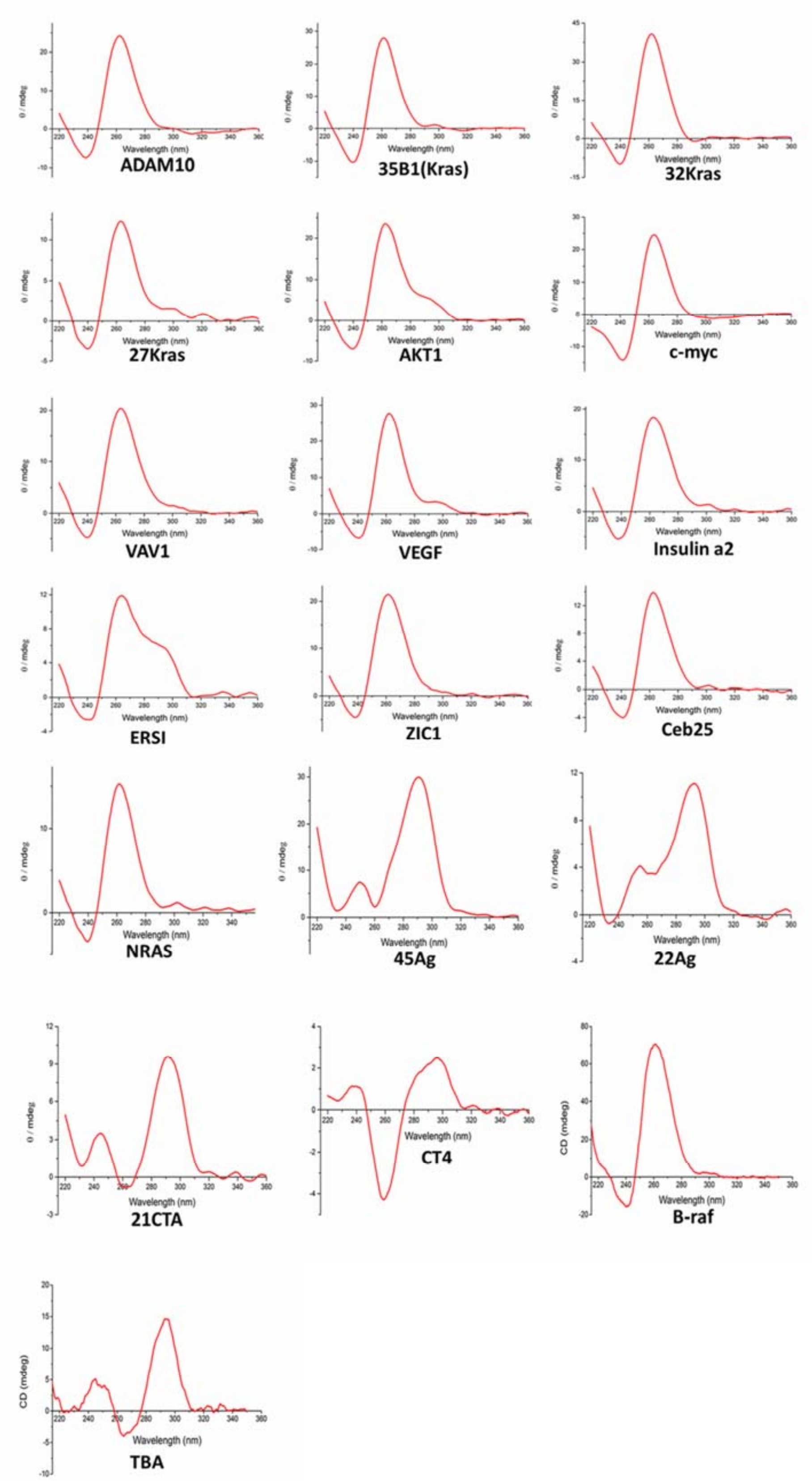

Figure S3. Circular dichroism spectra of 2 μM ThT-E with various DNA G-quadruplexes (2 μM) in 20

mM Tris-HCl (40 mM KCl, pH=7.4) solution.

Part 5 Spectrofluorimetric titration of the 22Ag

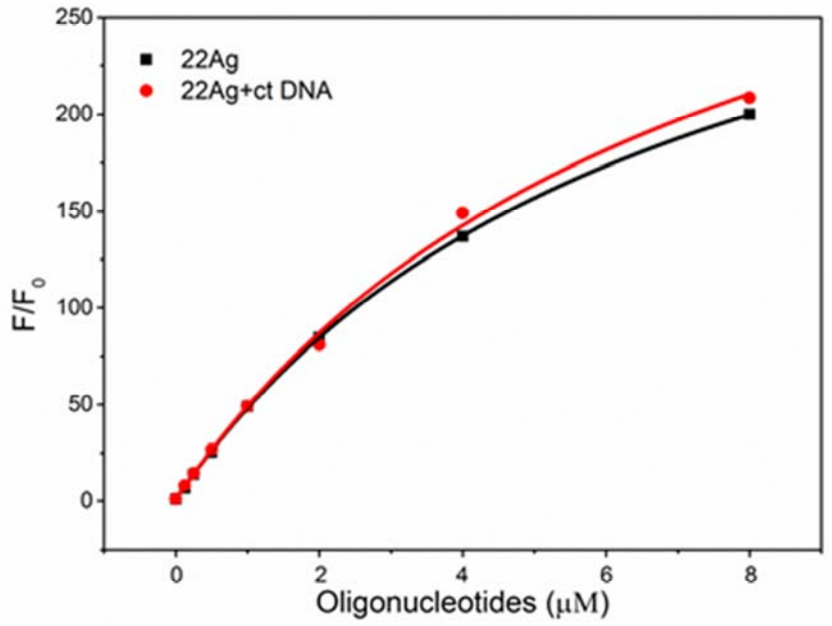

Figure S4. Spectrofluorimetric titration of the 22Ag to the ThT-E (2 μM) in the absence or presence of an excess of ct DNA (300 μg/mL).

Part 6 Job's plot analysis

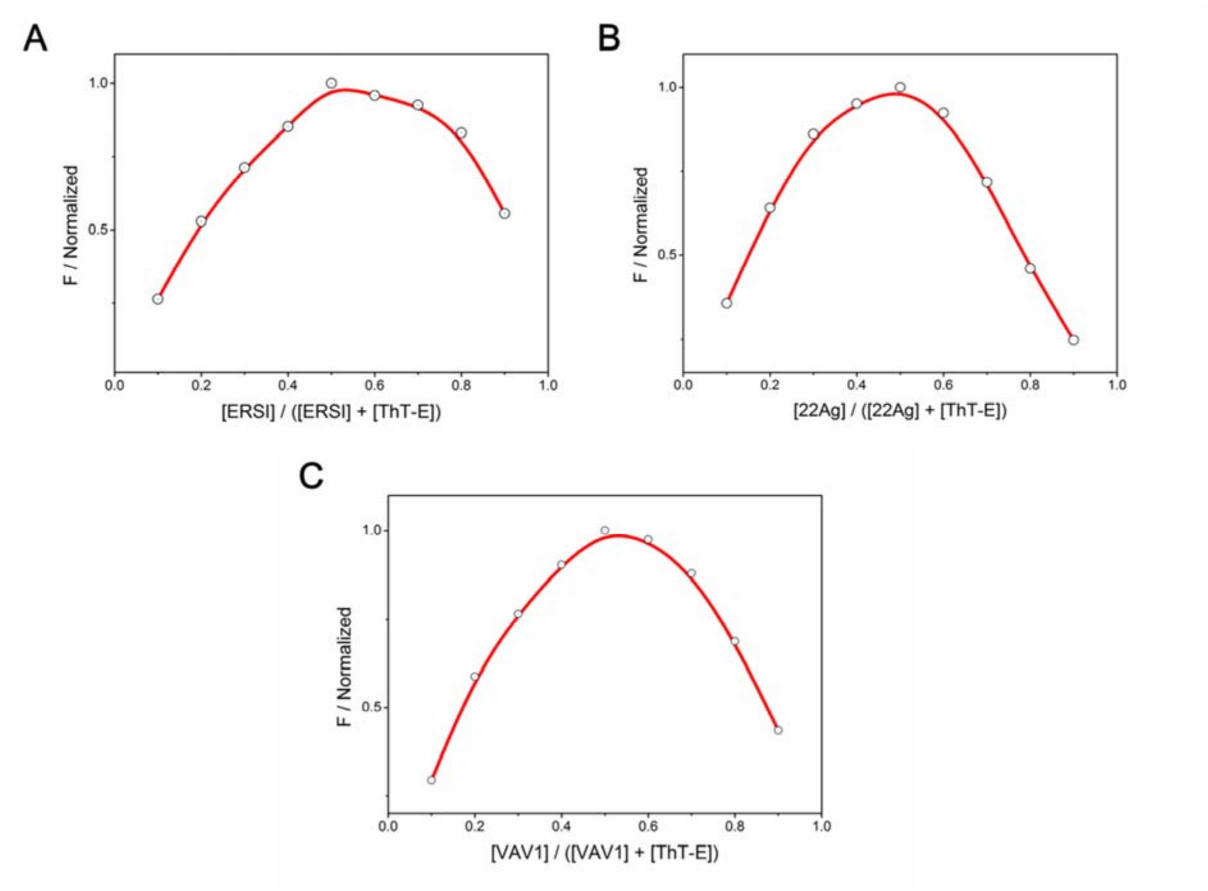

Figure S5. Job's plot analysis of the stoichiometry of G-quadruplexs binding to ThT-E (A) ERSI, (B) 22Ag and (C) VAV1. The total concentration of ThT-E and DNA G-quadruplex was kept constant at 4 μM.

Part 7 Determination of dissociation constants by ITC

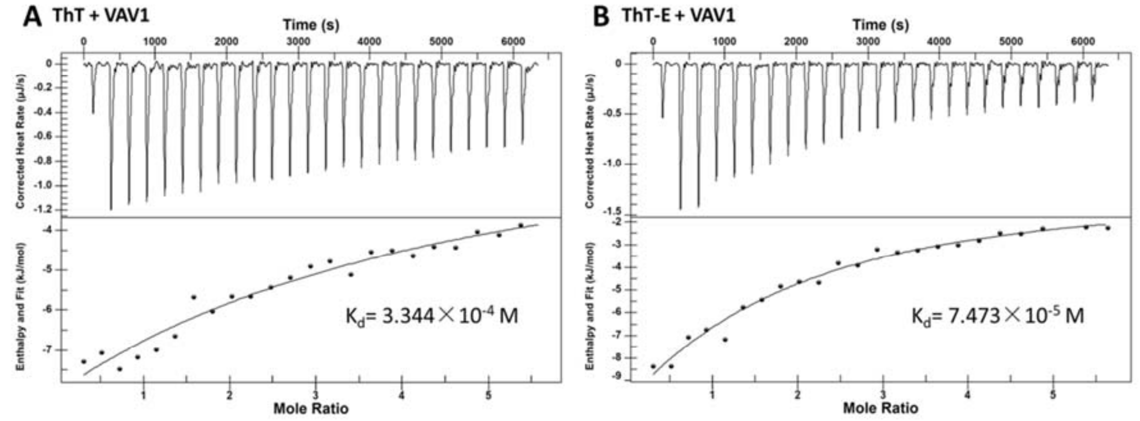

Figure S6. Representative results of G-quadruplexs with ThT (A) or ThT-E (B). Calorimetric analysis of the interaction of the G-quadruplexs (VAV1) with the ThT or ThT-E.

Part 8 UV illumination measurement

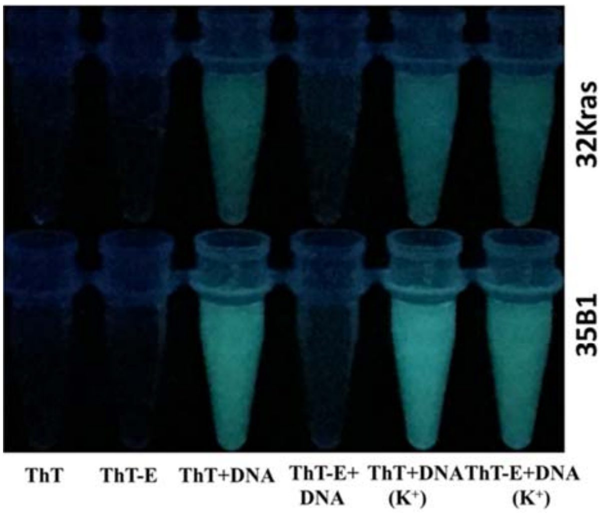

Figure.S7 Photograph of 2 μM 32Kras (top) or 35B1 (bottom) in 20 mM Tris-HCl (pH 7.4) solution under 254 nm UV light. From left to right were: ThT blank, ThT-E blank, DNA in ThT, DNA in ThT-E, DNA in ThT (40 mM K^+), DNA in ThT-E (40 mM K^+), respectively.

Part 9 The chemical shift's changes of the peaks of imino protons

Table S2. The chemical shift's changes of the peaks of imino protons of G7, G9, G13 and G16 with varying equivalents of ThT-E

ThT-E equivalents/ ∆δ (ppm)	7	16	9	13
0.5	0.0958	0.0889	0.0253	0.0312
1	0.1875	0.1903	0.0258	0.1043
2	0.2373	0.2251	0.039	0.1764

Part 10 Identification of ThT-E

a) Mass Spectrum

The calculated exact mass of $C_{20}H_{25}N_2S^+$ is 325.17.

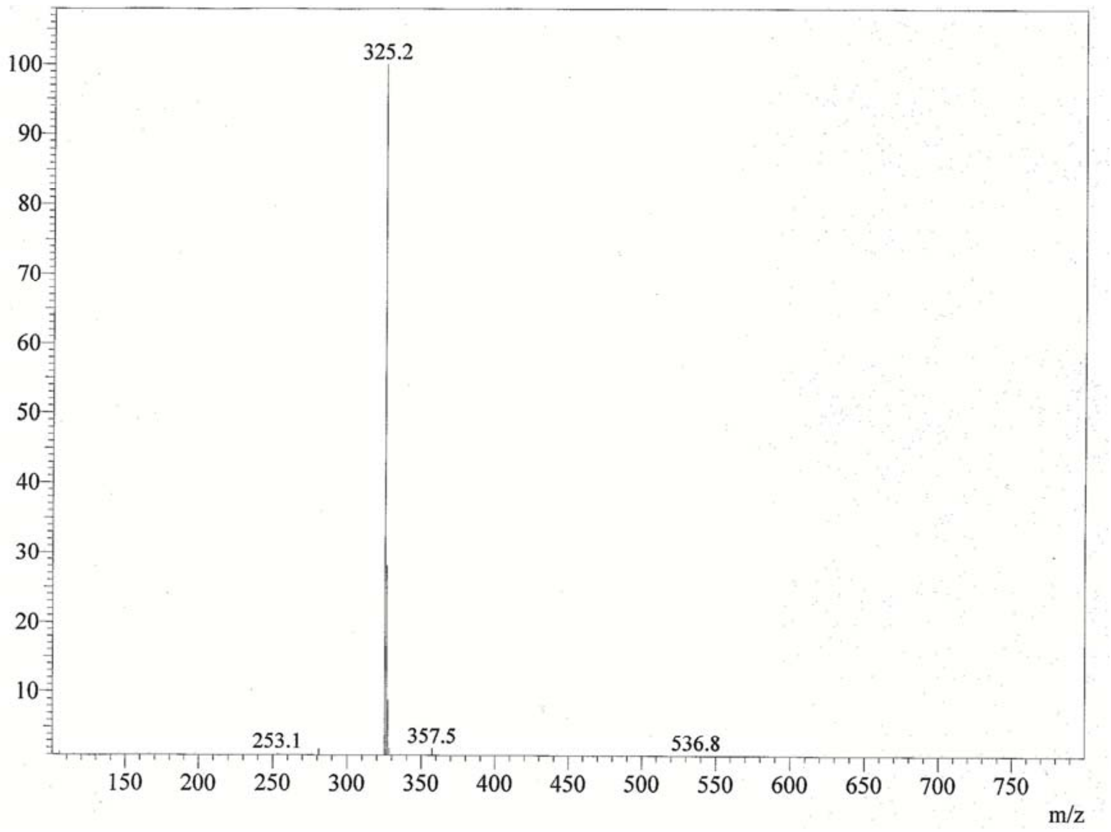

b) Elemental Analysis

Molecular formula of ThT-E: $C_{20}H_{25}IN_2S$

Element	N	C	H
Calculated Value（%）	6.19	53.10	5.57
Experimental Value (%)	5.91	52.26	5.57

c) NMR Spectrum

The numbering scheme of ThT-E

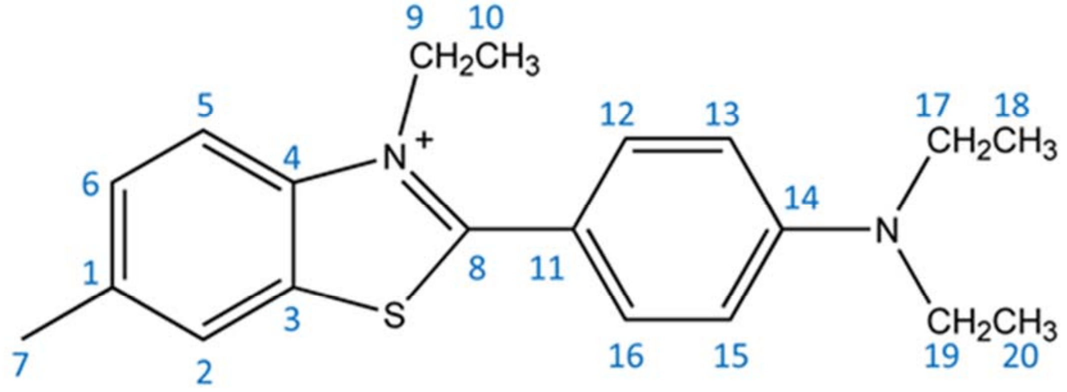

The ^{1}H-NMR spectrum of ThT-E in CD_3OD

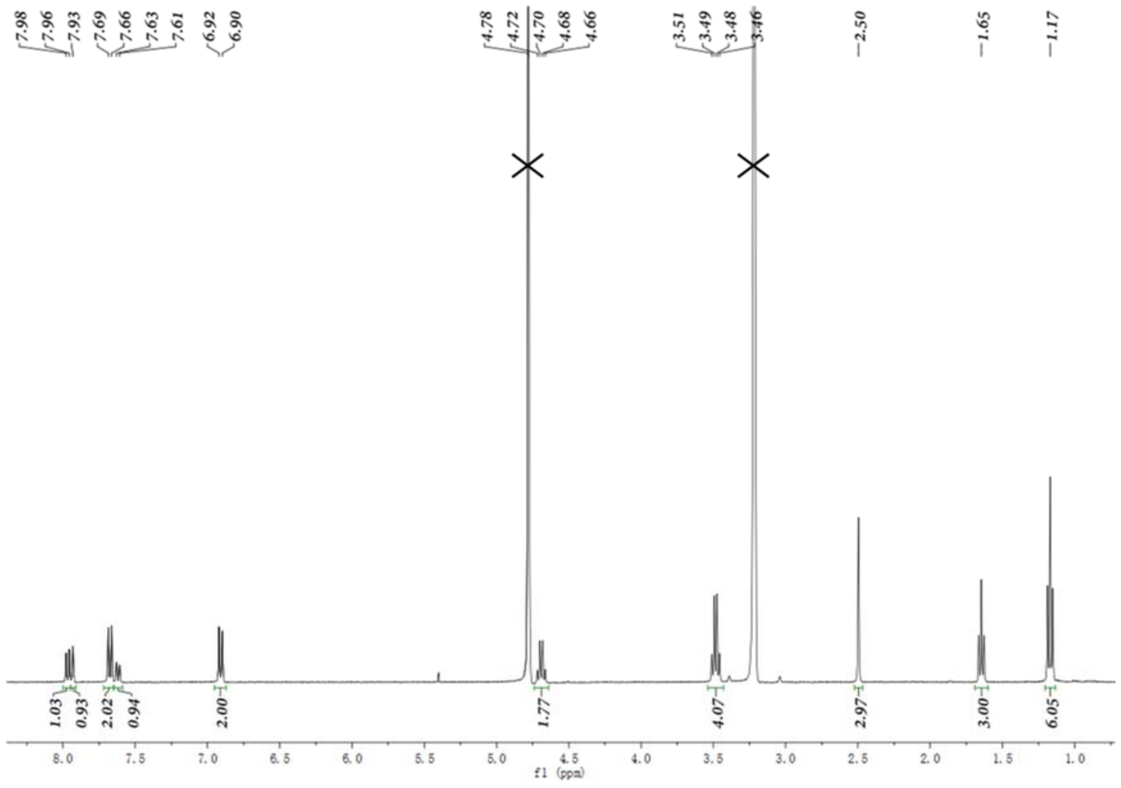

* The peaks of these protons are covered by the peak of the solvent CD_3OD

The full assignments of the proton peaks of ThT-E

Proton number	^{1}H peak	Proton number	^{1}H peak
1	--*	11	--*
2	7.93, s	12	7.69-7.66， d
3	--*	13	6.92-6.90, d
4	--*	14	--*
5	7.98-7.96, d	15	6.92-6.90, d
6	7.63-7.61, d	16	7.69-7.66， d
7	2.50, s	17	3.51-3.46, q
8	--*	18	1.17, t
9	4.72-4.66, q	19	3.51-3.46, q
10	1.65, t	20	1.17, t

The ^{13}C-NMR spectrum of ThT-E in CD_3OD

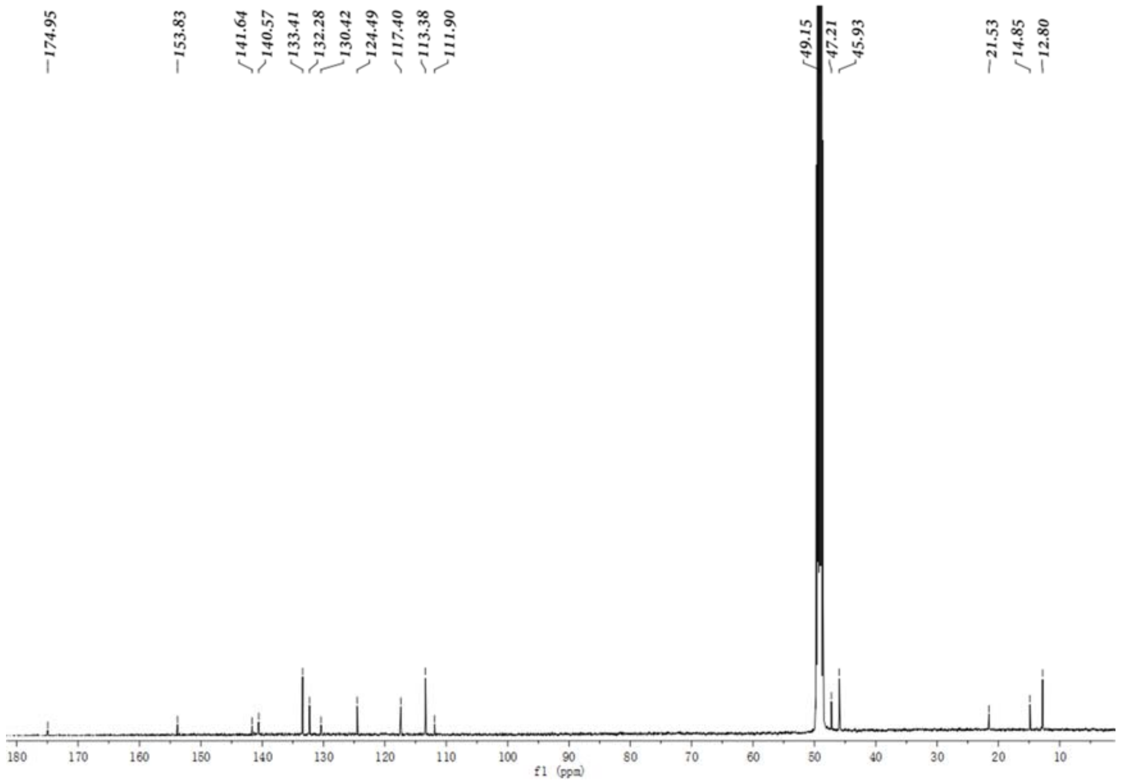

* The peaks of these ^{13}C are covered by the peak of the solvent CD_3OD

The full assignments of the ^{13}C peaks of ThT-E

Proton number	^{13}C peak	Proton number	^{13}C peak
1	140.57	11	111.90
2	124.49	12	113.38
3	130.42	13	133.41
4	141.64	14	153.83
5	117.40	15	133.41
6	132.28	16	113.38
7	21.53	17	45.93
8	174.95	18	12.80
9	47.21	19	45.93
10	14.85	20	12.80
